# A new genus and species of mandibulate nasute termite (Isoptera, Termitidae, Syntermitinae) from Brazil

**DOI:** 10.3897/zookeys.148.1278

**Published:** 2011-11-21

**Authors:** Mauricio M. Rocha, Eliana M. Cancello, Carolina Cuezzo

**Affiliations:** 1 Museu de Zoologia da Universidade de São Paulo, Cx. Postal 42.494, 04218-970 São Paulo, SP, Brasil; 2 CONICET- Instituto Superior de Entomología “Dr A. Willink”, Facultad de Ciencias Naturales e Instituto Miguel Lillo, UNT, Miguel Lillo 205, T4000JFE, San Miguel de Tucumán, Argentina

**Keywords:** Termite, *Acangaobitermes*, Syntermitinae, taxonomy

## Abstract

*Acangaobitermes krishnai* **gen. et sp. n.**, is described here, based on soldiers and workers collected in Brazil. Some characteristics suggest a close kinship with *Noirotitermes* Cancello & Myles, and both genera share the following traits absent in all other Syntermitinae: the microsculpturing on the soldier head capsule surface with internal granulations; the piercing mandibles with a single very reduced marginal tooth and the worker very similar in both genera. The most conspicuous differences between *Acangaobitermes* and *Noirotitermes* are the shape of the soldier head, the frontal tube and pronotum. The shape of the soldier head in *Noirotitermes* is unusual, with a very broad and short frontal tube, four conspicuous protuberances like sharp corners at the rear, while in the new genus the posterior contour of the head is devoid of these protuberances. The frontal tube of *Acangaobitermes* is elongate and conical, while in *Noirotitermes* it is short and very broad. The pronotum of *Acangaobitermes* is saddle-shaped as is usual in other Syntermitinae, while it is aberrant in *Noirotitermes*.

## Introduction

The “mandibulate nasutes” comprise a group of termite genera endemic to the Neotropical region. Fourteen genera are recognized within this group (*Armitermes* Wasmann, *Cahuallitermes* Constantino, *Cornitermes* Wasmann, *Curvitermes* Holmgren, *Cyrilliotermes* Fontes, *Embiratermes* Fontes, *Ibitermes* Fontes, *Labiotermes* Holmgren, *Macuxitermes* Cancello and Bandeira, *Noirotitermes* Cancello and Myles, *Paracurvitermes* Constantino and Carvalho, *Procornitermes* Emerson, *Rhynchotermes* Holmgren and *Syntermes* Holmgren), ranging from south of Mexico (*Cahuallitermes*) to northern Argentina (*Procornitermes*, *Syntermes*). The group is morphologically characterized by having soldiers with a large frontal gland opening, situated at the frontal tube apex, and functional mandibles.

In the past, the mandibulate nasutes were considered an ancestral group of Nasutitermitinae but recent studies highlighted an evolutionary history independent of true nasutes (Noirot 2001, Ohkuma et al. 2004, Inward et al. 2007). Engel and Krishna (2004) proposed Syntermitinae as a subfamily of Termitidae including four of the thirteen genera of mandibulate nasutes, and they affirm that the other mandibulate genera of Nasutitermitinae “may eventually be included” in the subfamily.

In this work we describe a new monotypic termite genus that seems to be closely related to *Noirotitermes*. Both genera share traits absent in all other Syntermitinae: the microsculpturing on the soldier head capsule surface with internal granulations; the piercing mandibles upturned, with a single reduced marginal tooth; and the worker almost identical in both genera.

## Materials and methods

The studied samples, including the holotype and paratypes, are in the Museum of Zoology of the University of São Paulo, São Paulo, Brazil (MZUSP). All comparisons with other syntermitine genera where based on data from taxonomic reviews or original descriptions [*Armitermes* (Rocha 2011), *Cahuallitermes* (Constantino 1994), *Labiotermes* (Constantino et al., 2006), *Macuxitermes* (Cancello and Bandeira 1992, Constantino 1997), *Noirotitermes* (Cancello and Myles 2000), *Paracurvitermes* (Constantino and Carvalho 2011) and *Syntermes* (Constantino 1995)] and examination of material in the MZUSP collection, that has specimens of all type species of syntermitine genera.

Terms used for pilosity are comparative: bristles are long erect setae with well-marked bases; hairs are shorter than bristles, less rigid and with inconspicuous bases; microscopic hairs are very short and visible only under at least 50 × magnification (not illustrated in the figures). Gut terminology follows Noirot (2001).

The morphometric characters used here and their correspondence with Roonwal’s system (Roonwal 1970) are indicated in parentheses as follows: length of head capsule, LH (9); width of head capsule, WH (18); length of frontal tube, LFT (28); length of left hind tibia, LT (85). All measurements were taken with a micrometric reticle.

Line drawings were made with a camera lucida, soldier photographs were obtained with a digital camera coupled to a stereomicroscope Leica M205C, and images of different depth of focus were further processed and merged with software. Worker mandibles were dissected and prepared for scanning electron microscopy. The worker enteric valve was mounted on Entellan (Merck) and photographed under an optic microscope. Scales are indicted in each illustration.

## Taxonomic treatment

### Acangaobitermes gen. n.

urn:lsid:zoobank.org:act:B72916A1-CF82-4685-BA8C-F48677C2A9B2

http://species-id.net/wiki/Acangaobitermes

#### Type species.

*Acangaobitermes krishnai* sp. n.

#### Etymology.

From Tupi, indigenous South American language, *acangaobi* meaning funneled head and the Latin *termes* meaning termite, in reference to soldier head capsule shape in profile. The name is masculine.

#### Description.

**Imago.** Unknown.

**Soldier.** Monomorphic. Head capsule sub-quadrangular with almost parallel lateral margins and two very discrete saliencies on the latero-posterior corners (Fig. 7, arrows). Surface of head capsule covered with numerous minute and closely set points of about equal diameter, forming a conspicuous and characteristic microsculpture (Figs 7–8). Frontal tube conical and upturned, in profile, apex with a relatively wide aperture surrounded by a white membrane. Antennae with 14 articles. Piercing slender mandibles; blade strongly curved inwards and upturned; a very small tooth at the base of the blade and a molar plate/prominence fully developed with no ridges. Clypeus very reduced. Labrum with a rounded and flat hyaline tip. Posmentum sub-rectangular with antero-lateral margins slightly concave. Coxae with a keel shape projection, pointing outwards and situated at distal antero-lateral margins (Fig. 2, arrow). Tibial spurs 2:2:2.

**Figures 1–4.**
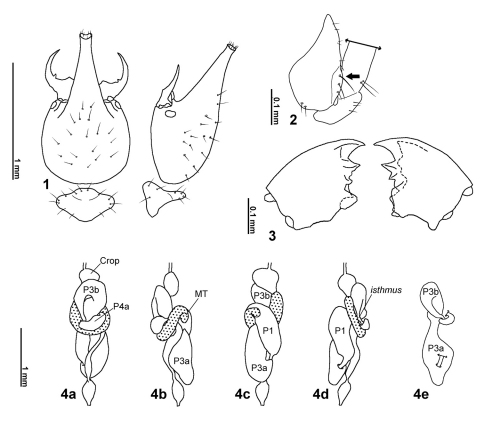
*Acangaobitermes krishnai* gen. et sp. n. **1** soldier head and pronotum in dorsal and profile view **2** soldier coxa in frontal view (arrow: keel shape projection) **3** worker mandibles **4** worker digestive tube *in situ*, a: dorsal view, b: right view, c: ventral view, d: left view and e: paunch in left view. MT= Mesenteric tongue; P1= first proctodeal segment (ileum); P3a and b = third proctodeal segment (paunch); P4a= first part of fourth proctodeal segment (colon).

**Figures 5–6. F2:**
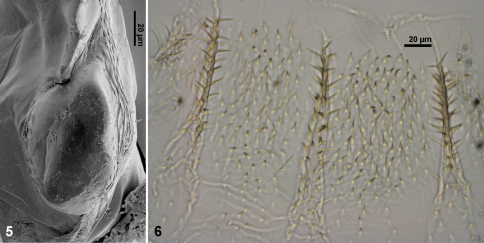
*Acangaobitermes krishnai* gen. et sp. n. **5** worker molar plate **6** enteric valve armature.

**Worker.** Monomorphic. Head capsule rounded. Postclypeusinflated. Antennae with 14 articles. Left mandible (Fig. 3): apical tooth larger than M1+2, margin between M1+2 and M3 sinuate, M3 distinct and smaller than M1+2, molar tooth conspicuous, partially hidden by molar prominence; molar prominence concave without ridges. Right mandible (Figs 3, 5): apical tooth larger than marginal teeth, M1 and M2 clearly distinct, molar plate concave without ridges (Fig. 5). Coxae smooth without projections. Body slender and elongated, digestive tube visible through abdominal sclerites. Tibial spurs 2:2:2.

Worker digestive tube. Crop asymmetrical, without any constriction separating it from gizzard. Cuticular armature of gizzard with 24 visible folds, six of first order, six of second and 12 of third; ratio between columnar and pulvillar belt approximately equal to one; pulvilli without armature or ornamentation. Mesenteron tubular. Short mixed segment present. Mesenteric tongue on the external side of the mesenteric arch, slightly strangled proximally. Two pairs of Malpighian tubules attached at the mesenteron-proctodeum junction, one internal side to the mesenteric arch and the other external. First proctodeal segment (P1), diagonal to body axis, more enlarged than mesenteron with about same size of proximal portion of paunch (P3a); distal end of P1 narrowed, forming a short neck prior to the attachment to P3. Enteric valve (P2) at the left side of the body. P3 slightly constricted between P3a and P3b. Dorsal torsion well-developed. P3 joined to colon (P4) on left side, isthmus short and parallel to body length. P4a dilated, U-Turn and P4b tubular.

##### Comparisons with other genera of Syntermitinae

Soldiers of *Syntermes*, *Cornitermes*, *Labiotermes*, and *Procornitermes* have a short frontal tube, not exceeding the labrum; a well-developed hyaline tip to the labrum; straighter cutting mandibles, with well-developed marginal teeth; and a larger body size. Soldiers of *Cahuallitermes* have straighter cutting mandibles, with well-developed marginal teeth; a well-developed hyaline tip to the labrum; and a larger body size. Soldiers of *Embiratermes* and *Ibitermes* have a larger body size, straighter and large mandibles, with well-developed marginal teeth in *Embiratermes* or totally absent in *Ibitermes*. Soldiers of *Cyrilliotermes* and *Curvitermes* have aberrant mandibles, with a molar plate, molar prominence and marginal teeth very similar to their corresponding worker mandibles; apical tooth fish-hooked in *Curvitermes*, reduced in *Cyrilliotermes*; and the frontal tube cylindrical and elongate in *Cyrilliotermes* (see more details of these two genera, including the dissected soldier mandibles, in Mathews 1997, page 226). *Paracurvitermes* has a broader head capsule with well developed conical and shorter frontal tube than *Acangaobiermes*; the mandibles are much longer, less curved with triangular teeth, very different from the new genus. The soldiers of *Rynchotermes* have strongly curved mandibles; a very long frontal tube; procoxae with a spine-like lateral projection; and a much larger body size. Soldiers of *Armitermes* have the pronotum, mesonotum, and metanotum with serrate lateral margins; mandibles with well-developed marginal teeth; and a larger body size (see Rocha, 2011, for a redescription and new illustrations of the genus). Lastly, the genus *Macuxitermes* has dimorphic soldiers, with an aberrant head shape; pronotum, mesonotum and metanotum with serrate lateral margins; and mandibles with well-developed marginal teeth.

Despite differences in the shape of the soldier head, *Acangaobitermes* shares many exclusive traits with *Noirotitermes*. The worker of *Acangaobitermes* is very similar to that of *Noirotitermes*, with same mandibular pattern, body size and shape (elongate), labrum and digestive tract, including the enteric valve armature. The worker differences between both genera are: the inner margin of apical teeth in left and right mandibles are much more concave in *Noirotitermes* than in the new genus; the M3 in left mandibleand M2 in right mandible both are larger in *Acangaobitermes* than in *Noirotitermes*; and the insertion of the enteric valve is in the body axis, while in *Noirotitermes* it is perpendicular to the body axis.

**Figure 7. F3:**
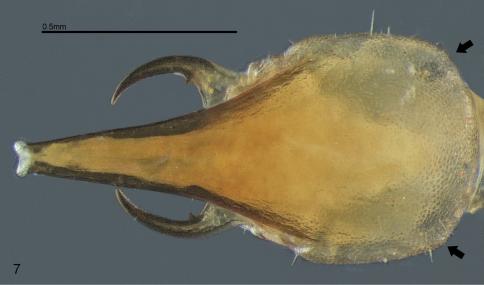
*Acangaobitermes krishnai* gen. et sp. n. Soldier head in dorsal view (Arrows: saliencies on latero-posterior margins).

**Figure 8. F4:**
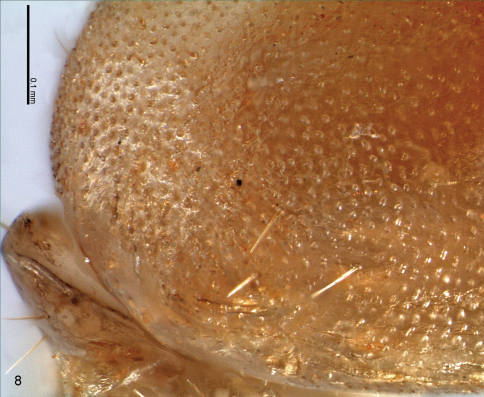
*Acangaobitermes krishnai* gen. et sp. n. Detail of the microsculputure of the soldier head capsule.

The soldiers of both species have the same microsculpturing on the head capsule surface and the internal granulations (Fig. 8), that are otherwise absent in all other species of Syntermitinae. The piercing, upturned mandibles, with a single marginal tooth reduced are nearly identical in both genera, while in all other Syntermitinae the marginal teeth are well-developed (or completely absent in *Ibitermes*). The two occipital saliencies are present in both genera and in *Macuxitermes*, but are much more discrete in *Acangaobitermes* (Fig. 7, arrows).

The most conspicuous differences between the new genus and *Noirotitermes* are the shapes of the soldier head and pronotum. The pronotum is aberrant in *Noirotitermes* and saddle-shaped in *Acangaobitermes*, as is usual for otherSyntermitinae. The shape of the soldier head in *Noirotitermes* is unusual, with a very broad frontal tube and two protuberances like sharp corners at the rear, while in the new genus the frontal tube is elongate and conical, similar to *Armitermes*, and the posterior rear part of the head is devoid of conspicuous projections.

### Acangaobitermes krishnai sp. n.

urn:lsid:zoobank.org:act:D82B4A68-0687-45E3-8275-AA24B9D4BFF3

http://species-id.net/wiki/Acangaobitermes_krishnai

Figs 1 [Fig F2] [Fig F3] [Fig F4] 9 

#### Holotype.

Soldier. Part of the lot MZUSP 13167, labeled “Parque Nac. Emas, GO, 22.iv.2004. Ninho 212, D. Costa col.” Kept separately in the same vial with paratypes.

#### Type-locality.

BRAZIL. Goiás: Parque Nacional das Emas (18°01.49'S; 52°57.87'W, 850 m).

#### Paratypes.

Soldiers and workers of MZUSP 13167 with same data as holotype. Goiás: Parque Estadual da Serra de Caldas Novas (17°44.7'S; 48°37.5'W, 1000 m), 23.iii.2008, D. E. Oliveira coll. (MZUSP 13168). Minas Gerais: Serra de São José (21°4.98'S; 44°10.02'W, 1250m), 11.iv.2007, E.M. Cancello coll. (MZUSP 11956). Rondônia: UHE Santo Antônio (8°50.63'S; 64°3.75'W, 100 m), 22.ix.2010, T. Carrijo & R. Santos coll. (MZUSP 13670).

#### Diagnosis.

As for the genus (*vide supra*).

#### Etymology.

Named in honor of Dr Kumar Krishna, for his important contributions to termite taxonomy.

#### Description.

**Imago.** Unknown.

**Soldier.** Shape of head, frontal tube, labrum, mandibles, pronotum under generic description. Antennae with 14 articles, 2nd half size of 1st, 3rd half of 2nd, 4th half of 3rd, 5th twice the size of 4th, subsequent articles sub-equal and similar to third. Scattered bristles, short hairs and microscopic hairs on top and lateral sides of head capsule, few bristles at rear portion. Frontal tube with microscopic hairs along its length and hairs around aperture of frontal tube. Pronotum with bristles on margins, plus two pairs of bristles at middle of anterior lobe. Mesonotum and metanotum with a row of bristles on posterior margins. Abdominal tergites and sternites with short hairs over surfaces and bristles on posterior margins. Head orange, mandibles ferruginous, body pale-yellow. Measurements, in millimeters, of four soldiers including the holotype: LH: 1.26–1.52; WH: 0.62–0.70; LFT: 0.56–0.74; LT: 0.64–0.66.

**Figure 9.**
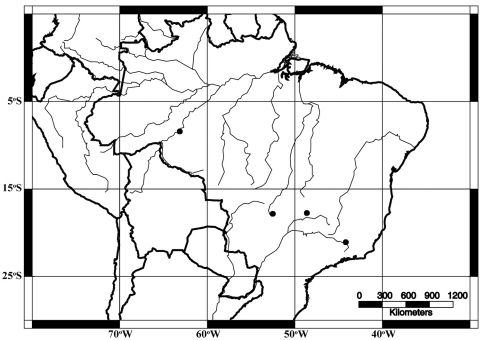
Geographic distribution of *Acangaobitermes krishnai*, gen. et sp. n.

**Worker.** External morphology under generic description. Head capsule with scattered bristles, antennae with some short hairs and sparse bristles, pronotum with bristles on margins and over surface of anterior lobe, mesonotum and metanotum with bristles on posterior margins. Abdominal tergites and sternites with short hairs over surfaces and bristles on posterior margins.

Digestive tube (Figs 4a–4e, 6). Coiling gut pattern and gizzard armature under generic description. P2 armature (Fig. 6) with three longitudinal equidistant cushions covered with strong and erect spines oriented perpendicular to gut contents flow, among the cushions minor spines settled at different orientations. P3 internally ornamented with long cuticular filaments (as described in Noirot 2001).

Biology. All the samples were collected in the soil or in nests of *Cornitermes cumulans* (Kollar) and *Armitermes euamignathus* Silvestri, in areas of openformation. The specimens from state of Goiás are collected in a Cerrado formation. From the state of Rondônia in a border line between primary forest and pasture. From Minas Gerais state in a “Campo rupestre”, a characteristic altitudinal field, with granitic outcrops and composed by xeric vegetation.

## Discussion

Relationships among the mandibulate nasute genera are not yet clear, despite considerable evidence that they are a monophyletic group (e.g., Inward et al., 2007). Rocha (2011) conducted a taxonomic revision and a phylogenetic analysis of the genus *Armitermes*, including all type species of the genera of Syntermitinae. This analysis supports the hypothesis that *Macuxitermes*, *Acangaobitermes*, and *Noirotitermes* form a monophyletic group and that the last two are most closely related, with the occipital saliencies and the type of folds and their arrangement on the enteric valve as synapomorphies for the three genera.

## Supplementary Material

XML Treatment for Acangaobitermes

XML Treatment for Acangaobitermes krishnai
